# Micro-Longitudinal Examination of Emotion Dysregulation and Posttraumatic Stress Disorder Symptoms among Community Women Experiencing Intimate Partner Violence: Modeling Reciprocal Relationships Using Dynamic Structural Equation Modeling

**DOI:** 10.55913/joep.v1i1.23

**Published:** 2023-05-27

**Authors:** Nicole H. Weiss, Alexa M. Raudales, Ateka A. Contractor, Shannon R. Forkus, Reina Kiefer, Leslie A. Brick, Tami P. Sullivan

**Affiliations:** 1 University of Rhode Island, Department of Psychology, USA; 2 University of North Texas, Department of Psychology, USA; 3 Brown University, Department of Psychiatry and Human Behavior, USA; 4 Yale University, Department of Psychiatry, USA

**Keywords:** emotion dysregulation, negative emotions, positive emotions, posttraumatic stress disorder, intimate partner violence, dynamic structural equation modeling

## Abstract

Research examining emotion dysregulation and posttraumatic stress disorder (PTSD) has seen tremendous growth over the past decade. However, past investigations have almost exclusively relied on cross-sectional designs and have neglected to consider the potential role of dysregulation stemming from positive emotions. The current study utilized rigorous methodology (experience sampling) and statistics (dynamic structural equation modeling) to explicate daily reciprocal associations between negative and positive emotion dysregulation and PTSD symptoms. Participants were 145 community women (M age = 40.66, 40.7% white) experiencing intimate partner violence (IPV) and using substances who participated in a baseline interview and then completed surveys three times a day for 30 days. Results at the between-person level showed that women who reported higher negative and positive emotion dysregulation also reported more PTSD symptoms. At the within-person level, findings supported a significant contemporaneous effect between positive emotion dysregulation and PTSD symptoms. Further, there was a significant cross-lagged effect from negative emotion dysregulation to next-interval PTSD symptoms. Results suggest that positive emotion dysregulation co-occurs with PTSD symptoms and that negative emotion dysregulation predicts PTSD symptoms. Findings provide additional support for the utility of addressing both negative and positive emotion dysregulation in the treatment of PTSD among women experiencing IPV.

Intimate partner violence (IPV) is a pervasive and devastating public health concern among women, with nearly 1 in 3 women reporting experiences of IPV during their lifetime (Smith et al., 2018). Posttraumatic stress disorder (PTSD) is one common consequence of IPV among women, with meta-analytic review identifying a weighted mean prevalence for PTSD of 63.8% (range: 31% to 84%) among women who had experienced IPV (Golding, 1999). As described in the fifth edition of the Diagnostic and Statistical Manual of Mental Disorders (DSM-5; American Psychiatric Association, 2013), PTSD is etiologically tied to trauma (such as IPV) and characterized by intrusions (e.g., nightmares, flashbacks), avoidance of internal (e.g., thoughts, feelings) and external (e.g., people, places) trauma-related cues, negative alterations in cognitions and mood (e.g., blaming self or others, negative emotions), and alterations in arousal and reactivity (e.g., hypervigilance, exaggerated startle). Among women who have experienced IPV, PTSD leads to serious and widespread consequences for health and well-being (Hellmuth et al., 2014; Laffaye et al., 2003; Stein & Kennedy, 2001; Woods et al., 2008). Thus, research that improves our understanding of PTSD among women experiencing IPV is clinically significant.

A fast-growing body of literature has linked emotion dysregulation to PTSD (for meta-analytic review, see Seligowski et al., 2015), including among women experiencing IPV (Weiss, Darosh, et al., 2018; Weiss, Dixon-Gordon, et al., 2018; Weiss, Nelson, et al., 2019). As defined by Gratz and Roemer (2004), emotion dysregulation is a multi-faceted construct involving: (a) lack of awareness, understanding, and acceptance of emotions; (b) an inability to control behaviors when experiencing emotions; (c) limited access to situationally appropriate strategies for modulating emotions; and (d) an unwillingness to experience emotions as part of pursuing meaningful activities in life (see also Gratz & Tull, 2010). PTSD has been purported to interfere with the ability to effectively regulate emotions. One explanation for this is that PTSD symptoms are associated with more intense negative emotions (DiMauro et al., 2016), and more intense negative emotions are more difficult to regulate (Salsman & Linehan, 2012). For instance, greater intensity of negative emotions may deplete capacities for regulation (Inzlicht & Schmeichel, 2012), leading people to implement emotion regulation strategies that are less effective in the long-term (e.g., suppression; Dixon-Gordon et al., 2015). Further, individuals may have greater difficulty controlling behavior (e.g., impulsive or goal-directed) in the context of intense negative emotions elicited by PTSD symptoms because they display a non-accepting, evaluative stance toward these emotions (Weiss, Schick, et al., 2019; Weiss et al., 2012; Weiss, Walsh, et al., 2019). In line with these suggestions, women experiencing IPV with PTSD report higher emotion dysregulation compared to those without PTSD (Weiss, Dixon-Gordon, et al., 2018), and higher emotion dysregulation has been related to greater PTSD symptom severity among women experiencing IPV (Weiss, Nelson, et al., 2019).

An important limitation of the extant research in this area is its near-exclusive reliance on cross-sectional designs (see Seligowski et al., 2015), precluding determination of the temporal ordering of the relation between emotion dysregulation and PTSD. Longitudinal studies in this area are scarce (Bardeen et al., 2013; Boden et al., 2013), and, in addition to being subject to memory decay/distortion and heuristic (e.g., availability) bias (Shiffman et al., 2008; Stone & Shiffman, 1994; Stone et al., 2007)—particularly relevant to investigation of emotions (Shiffman et al., 2008)—do not allow for identification of proximal reinforcers. Nonetheless, early prospective findings suggest that emotion dysregulation and PTSD symptoms may reciprocally influence one another (e.g., Bardeen et al., 2013; Rooney et al., 2022; Weiss, Walsh, et al., 2019). For instance, Bardeen et al. (2013) examined emotion dysregulation and PTSD symptoms at three time-points: prior to a mass shooting (T1), in the acute aftermath of the shooting (T2), and approximately eight months after the shooting (T3). Emotion dysregulation was found to prospectively predict PTSD symptoms from T1 to T2 and T2 to T3, and PTSD symptoms were found to prospectively predict emotion dysregulation from T1 to T2. Consistent with these findings, theoretical explanations also posit an effect of emotion dysregulation on subsequent PTSD. The presence of elevated emotion dysregulation may result in greater appraisals of threat and more intense emotional responses, factors linked to the exacerbation of PTSD symptoms (Bovin & Marx, 2011). Furthermore, individuals with heightened emotion dysregulation may have limited access to strategies for effectively down-regulating trauma-related symptoms and distress (Tull et al., 2007; Weiss et al., 2013). In turn, they may prioritize tactics that help them to escape or avoid trauma-related experiences, thereby preventing exposure to corrective information and interfering with emotional processing (Foa & Kozak, 1986). Collectively, these findings suggest a reciprocal association between emotion dysregulation and PTSD, such that PTSD symptoms predict later emotion dysregulation and emotion dysregulation predicts later PTSD symptoms.

Experience sampling methods (ESM) are one promising method for empirically testing this hypothesis. ESM involve repeated within-day surveying of real-world experiences in near real-time, allowing researchers to capture processes that fluctuate over time (Stone & Shiffman, 2002). ESM is less subject to memory decay/distortion and heuristic (e.g., availability) bias (Shiffman et al., 2008; Stone & Shiffman, 1994; Stone et al., 2007), factors particularly relevant to the study of emotional processes (Shiffman et al., 2008), and thus strengthens the ecological validity of research findings. Moreover, by providing data on both between- and within-person relations, ESM averts the problem of ecological fallacy where data, because they have been aggregated for a group, misrepresent the experiences of individuals within that group (Robinson, 2009). To analyze ESM data in the current study, Dynamic Structural Equation Modeling (DSEM; Asparouhov et al., 2018), a cutting-edge statistical approach that combines time-series level data for several subjects concomitantly to model associations among within-person variables over time, will be employed. DSEM addresses drawbacks of traditional multilevel approaches that limit their utility for analyzing ESM data—it takes nonequidistant observations into account; models random effects for both predictor and outcome variables as well as lagged effects using latent variables; and separates within- and between-person effects in a single model (Falkenström et al., 2017; Ramseyer et al., 2014). Use of sophisticated methods (ESM) and analytics (DSEM) will considerably advance our understanding of the link between emotion dysregulation and PTSD by clarifying its potentially reciprocal nature at the momentary level and in the real world.

Of note, we are not aware of any investigations using ESM to examine the association between emotion dysregulation and PTSD. We were able to identify one ESM study that explored the relation between emotion regulation strategies and PTSD symptoms (Short et al., 2018). Findings indicated that earlier maladaptive emotion regulation strategies increased PTSD symptoms later in the day; earlier adaptive emotion regulation strategies did not predict later PTSD symptoms. Importantly, whether earlier PTSD symptoms predicted emotion regulation strategies later in the day was not explored. Further, while interconnected, emotion dysregulation and emotion regulation strategies are unique processes (Tull & Aldao, 2015). Specifically, whereas emotion dysregulation captures the typical or dispositional ways in which individuals understand, regard, and respond to their emotional experiences—or their emotion regulation potential or abilities—emotion regulation strategies refer to the specific tactics individuals use (e.g., reappraisal, suppression) to influence the experience and expression of their emotions (Gross, 2015). The type of emotion regulation strategies implemented in a given situation—and their ultimate success—is influenced by emotion dysregulation. For example, an individual who is less accepting of emotions may be more likely to use emotional avoidance in the context of distress. However, this relationship is bidirectional, with emotion dysregulation also being impacted by the types of emotion regulation strategies an individual uses. For instance, strategies that serve an emotionally avoidant function may lead to less acceptance of emotions. Research using ESM to examine the link between emotion dysregulation and PTSD symptoms is needed.

Addressing another important limitation of existing research, the current study will measure dysregulation stemming from both negative and positive emotional experiences. Recent literature indicates that some individuals experience positive emotion dysregulation, including nonaccepting responses to positive emotions and behavioral dyscontrol (e.g., impulsivity) in the context of positive emotions (Weiss, Darosh, et al., 2019; Weiss, Gratz, et al., 2015). Notably, positive emotion dysregulation may be particularly salient to PTSD. For instance, individuals with PTSD may exhibit fear of physiological arousal (Raudales et al., 2021) and competing negative cognitions (Frewen et al., 2012) in the context of positive emotions that drive nonacceptance of positive emotions. Behavioral dyscontrol may stem from a reduced capacity to inhibit impulsive or reward-seeking behaviors in the context of positive emotions among individuals with PTSD (Weiss, Tull, et al., 2015). Consistent with these theoretical accounts, early empirical evidence provides support for an association between positive emotion dysregulation and PTSD, including among women experiencing IPV. Women experiencing IPV with versus without PTSD, as well as those with greater severity of PTSD symptoms, report heightened levels of positive emotion dysregulation (Weiss, Dixon-Gordon, et al., 2018). Moreover, among women experiencing IPV, positive emotion dysregulation appears to have incremental utility, beyond negative emotion dysregulation, in predicting PTSD symptom severity (Weiss, Nelson, et al., 2019). Relatedly, the presence of positive emotion dysregulation among women experiencing IPV has been found to be a particularly potent risk factor for PTSD compared to negative emotion dysregulation alone (Weiss, Darosh, et al., 2018). These findings underscore the need for examining both negative and positive emotion dysregulation in relation to PTSD among women experiencing IPV.

The current study utilized state-of-the-art methodology and statistics to examine the daily reciprocal relations between both negative and positive emotion dysregulation and PTSD symptoms in a sample of community women experiencing IPV. We hypothesized that both negative and positive emotion dysregulation and PTSD symptoms would co-occur within intervals (contemporaneous effects). Further, we expected that both negative and positive emotion dysregulation will predict next-interval PTSD symptoms and that PTSD symptoms would predict next-interval negative and positive emotion dysregulation (cross-lagged effects).

## Method

### Participants

Recruitment materials were posted in community establishments throughout Providence County, Rhode Island including grocery stores, laundromats, and shops; selected state offices such as the Office of Housing and Community Development; and waiting rooms, bathrooms, and exam rooms of urban-area primary care clinics; as well as in website postings (e.g., Craigslist). Eligibility was determined through self-report during phone screen. Participants were women who had experienced physical or sexual victimization in the past 30 days by their current male partner and used any amount of drugs or alcohol during that time. Additional inclusion criteria were: (1) age 18 or older, (2) fluent in the English language, and (3) current involvement in a relationship of at least six months’ duration with contact at least twice a week. Exclusion criteria were (a) current mania/psychosis (assessed in the baseline session with the Structured Clinical Interview for DSM-V [SCID-5]) (First & Williams, 2016), (b) current impairment in cognitive functioning (assessed in the baseline session using the Mini-Mental Status Exam and requiring a score > 24) (Folstein et al., 1975), (c) self-reported current pregnancy, (d) colorblindness, (e) cardiovascular disease, and (f) residence in a shelter or group home. The final sample included 145 women who participated in the baseline session and completed at least one survey during the ESM period (see Procedures); demographic characteristics are summarized in Table 1.

### Procedures

All procedures were reviewed and approved by the University of Rhode Island Institutional Review Board. This is a secondary data analysis from a larger study examining the proximal role and temporal ordering of emotion dysregulation in substance use and HIV/sexual risk, and the role of PTSD (Weiss, Brick, Schick, et al., 2022; Weiss et al., in press). The larger study entailed (a) a baseline session, (b) an experimental session, (c) 30 days of ESM using interactive voice recording (IVR) technology, and (d) a follow-up session. The current study used data from the baseline session and ESM period. Participants were provided with a list of community resources. Assistance with referrals was provided upon participant request. The principal investigator, a licensed psychologist in the state of Rhode Island, was available on-call if participants required additional mental health support.

### Baseline Session.

Baseline sessions were conducted by a female bachelors- or masters-level clinical psychology doctoral student in a private office to protect participants’ safety and confidentiality. After providing informed consent, participants were interviewed using a structured diagnostic assessment and then answered self-report measures on a computer. Participants were compensated with $40 for completing the baseline session.

### ESM Period.

During the ESM period, participants completed surveys through the IVR telephone system three times a day for 30 days. Calls could be initiated by the participant or the participant could have elected to have the IVR system initiate a call. Regarding the latter option, calls were individualized to the participant (e.g., based on their typical schedule). Surveys took place between 4:00 a.m. and 11:59 a.m. (morning), 12:00 p.m. and 5:59 p.m. (afternoon), and 6:00 p.m. and 3:59 a.m. (evening). An example call schedule for someone who elected to have the IVR system initiate a call may be 9am (morning), 2pm (afternoon), and 8pm (evening). For each survey, participants were asked to report on experiences since the previous reporting period. Participants were trained to use the IVR telephone system to record their information daily during the experimental session, with training procedures modeled after those detailed in Stone and Shiffman (2002). Participants were compensated $1 for each completed survey as well as received weekly bonuses of $5 if > 80% of the ESM surveys had been completed.

### Measures

#### Demographic Information.

Participants reported their age, gender, racial and ethnic background, educational level, employment status, and income level.

#### Diagnostic Measure.

A computerized version of the SCID-5 was administered to establish current PTSD diagnosis (First & Williams, 2016). The SCID-5, a gold standard semi-structured assessment instrument for psychiatric disorders, has been found to yield valid and reliable current and lifetime diagnoses across several psychiatric disorders, including current PTSD. There is evidence of moderate to excellent interrater reliability across major diagnostic categories, including sensitivity of 1.00, specificity of 0.96, and a kappa of 0.80 for PTSD (Osório et al., 2019). SCID-5 interviews were conducted by clinical psychology doctoral students trained to reliability with the principal investigator. All data were reviewed by the principal investigator. In the case of ambiguous responses, data were discussed by the principal investigator and interviewer until a consensus was reached.

### Daily Measures.

#### Negative Emotion Dysregulation.

The Momentary Difficulties in Emotion Regulation Scale (mDERS; Weiss et al., 2021), an abbreviated, six-item momentary version of the Difficulties in Emotion Regulation Scale (DERS; Gratz & Roemer, 2004), assessed negative emotion dysregulation since the prior reporting interval across six domains: nonacceptance of negative emotions, difficulties engaging in goal-directed behaviors when experiencing negative emotions, difficulties controlling impulsive behaviors when experiencing negative emotions, limited access to emotion regulation strategies perceived as effective in the context of negative emotions, lack of negative emotional awareness, and lack of negative emotional clarity. The DERS is widely used and has sound psychometric properties (Gratz & Roemer, 2004). Participants rated the extent to which items applied to them using a 4-point Likert-type scale ranging from 1 (not true at all) to 4 (very true). Items were summed; higher scores indicated greater momentary difficulties regulating negative emotions. Multilevel reliabilities (Geldhof et al., 2014) in the current sample were good (within-person ω = 0.83, between-person ω = 0.98).

#### Positive Emotion Dysregulation.

The Momentary Difficulties in Emotion Regulation Scale – Positive (mDERS-P; Weiss et al., 2021), an abbreviated, six-item momentary version of the Difficulties in Emotion Regulation Scale – Positive (DERS-P; Weiss, Gratz, et al., 2015), was utilized to assess positive emotion dysregulation since the prior reporting interval across six domains: nonacceptance of positive emotions, difficulties engaging in goal-directed behaviors when experiencing positive emotions, difficulties controlling impulsive behaviors when experiencing positive emotions, limited access to emotion regulation strategies perceived as effective in the context of positive emotions, lack of positive emotional awareness, and lack of positive emotional clarity. The DERS-P has acceptable reliability and validity (Weiss, Darosh, et al., 2019; Weiss, Gratz, et al., 2015). Participants rated the extent to which items applied to them using a 4-point Likert-type scale ranging from 1 (not true at all) to 4 (very true). Items were summed; higher scores indicated greater momentary difficulties regulating positive emotions. Multilevel reliabilities (Geldhof et al., 2014) in the current sample were good (within-person ω = 0.78, between-person ω = 0.98).

#### Posttraumatic Stress Symptoms.

A modified version of the PTSD Checklist for the DSM-5 (PCL-5; Weathers et al., 2013) was used to assess momentary PTSD symptoms corresponding to the DSM-5 criteria (APA, 2013). The PCL-5 has excellent psychometric properties (Bovin et al., 2016; Wortmann et al., 2016). The modified version included seven dichotomized (i.e., yes/no) items (e.g., “Did you try to avoid things that reminded you of the experience, such as specific thoughts, feelings, people, places, activities, or situations?”). These items correspond with the 7-factor Hybrid model of PTSD, which has the most empirical support (Armour et al., 2015). Specifically, participants were asked to respond based on their worst trauma and indicate whether or not they have experienced each symptom cluster since the previous survey (i.e., re-experiencing, avoidance, negative alterations in cognitions and mood, alterations in arousal and reactivity, negative affect, anhedonia, externalizing behaviors, anxious arousal, and dysphoric arousal). Items were summed, with higher scores indicative of greater PTSD symptoms. Multilevel reliabilities (Geldhof et al., 2014) in the current sample were good (within-person ω = 0.73, between-person ω = 0.94).

### Analytic Strategy

Analyses were conducted in Mplus Version 8.6 (Muthen & Muthen, 1998-2017). To examine the reciprocal relations between emotion dysregulation and PTSD, we modeled data using the DSEM framework (Tihomir Asparouhov et al., 2018; Asparouhov & Muthén, 2020). Data were fit using Bayesian estimation with a minimum of 5,000 iterations and diffuse priors. Model convergence was determined using the Potential Scale Reduction (PSR), with values close to one indicative of stable convergence. A significant non-zero parameter was determined using 95% credibility intervals (CIs) produced by the Bayesian estimation (Asparouhov & Muthén, 2020). Missing data were sampled from their conditional posterior, thereby taking into account the autocorrelation structure of the individual’s data (Hamaker et al., 2018).

Cross-lagged and autoregressive effects were tested concurrently. Negative and positive emotion dysregulation were examined in separate models. To test the cross-lagged relations between emotion dysregulation and PTSD symptoms, lag 1 (i.e., first-order) vector autoregressive (VAR[1]) effects were modeled. Specifically, emotion dysregulation for person i at time t ($〖\text{ED}〗\;\_{\text{it}}^{\wedge }((W))$) was regressed on lag-1 PTSD ($〖\text{PTSD}〗\;\_{\left(\text{it-}1\right)}^{\wedge }((W))$); with cross-lag parameter, ϕ_(EP,i)) and lag-1 emotion dysregulation ($〖\text{ED}〗\;\_{\left(\text{it-}1\right)}^{\wedge }((W))$); with autoregressive parameter, ϕ_(EE,i)). Similarly, PTSD for person i at time t ($〖\text{PTSD}〗\;\_{\text{it}}^{\wedge }((W))$) was regressed on lag-1 emotion dysregulation ($〖\text{ED}〗\;\_{\left(\text{it-}1\right)}^{\wedge }((W))$); with cross-lag parameter, ϕ_(PE,i)) and lag-1 PTSD ($〖\text{PTSD}〗\;\_{\left(\text{it-}1\right)}^{\wedge }((W))$); with autoregressive parameter ϕ_(PP,i)). $〖\text{ED}〗\;\_{\text{it}}^{\wedge }((W))=\phi \_(\text{EE},i)\;〖\text{ED}〗\;\_{\left(\text{it-}1\right)}^{\wedge }((W))+\phi \_(\text{EP},i)\;〖\text{PTSD}〗\;\_{\left(\text{it-}1\right)}^{\wedge }((W))+\zeta \_(\text{ED},\text{it})\;〖\text{PTSD}〗\;\_{\text{it}}^{\wedge }((W))=\phi \_(\text{PP},i)\;〖\text{PTSD}〗\;\_{\left(\text{it-}1\right)}^{\wedge }((W))+\phi \_(\text{PE},i)\;〖\text{ED}〗\;\_{\left(\text{it-}1\right)}^{\wedge }((W))+\zeta \_(\text{PTSD},\text{it})$

Also represented in these equations are the residuals (ζ_(ED,it),ζ_(PTSD,it)), or innovations, which may covary across individuals and time. Moreover, this modeling allows for the deconstruction of within-person means of emotion dysregulation and PTSD, which can be regarded as an individual’s average scores, as well as temporal fluctuations from these values, which can be regarded as an individual’s momentary scores (Tihomir Asparouhov et al., 2018; Hamaker et al., 2018; McNeish & Hamaker, 2019). Autoregression parameters, commonly referred to as inertia, indicate how quickly an individual returned to their average score after being perturbed, with values further away from zero indicating a slower return. Cross-lagged parameters (i.e., spill-over) indicate the effect at a specific interval of one variable to another. During model building, we took an iterative and systematic approach. First, we began by fitting a simple model in which only fixed effects were estimated. Next, we included random effects for the autoregressions and cross-lagged effects. Then, we included random effects for each residual variance. Finally, we included random effects for the covariance between emotion dysregulation and PTSD symptoms. At each step we examined the model to evaluate model fit, whether convergence had been met, and whether random effects were significant. If a model did not converge upon addition of a random effect, we proceeded with model testing by only estimating fixed effects for that parameter.

This study is a secondary analysis and therefore power was not conducted a priori. However, based on the results of a simulation study (Schultzberg & Muthen, 2018), with a sample size of N=145 and t=90 possible surveys per person (maximum of 13,050 observations), we are well within the recommended sample size recommendations for good performance of a model with random effects estimated for the mean, autoregression, and residual variance.

## Results

### Preliminary Analyses

Participants (N = 145) completed, on average, 64.90 surveys (SD = 28.12) out of 90, for a compliance rate of 72.1%, and provided a total of 11,506 observations. Within-person average scores on negative and positive emotion dysregulation across the ESM period were 5.85 (SD = 0.37) and 5.68 (SD = 0.36), respectively. All participants reported PTSD symptoms on at least one day during the ESM period (M days = 47.16, SD = 29.37). Over a third of the sample (n = 53, 36.6%) met diagnostic criteria for current PTSD using the SCID-5.

### Negative Emotion Dysregulation

After model testing, a model in which random effects were estimated for the means, autoregressions, cross-lags, and residual variances (but no random effect for the covariance) converged with PSR values of 1.003 after 5,000 iterations. The fixed and random effects are presented in Table 2. Significant positive fixed and random cross-lagged effects were found for negative emotion dysregulation and next-interval PTSD symptoms (Standardized Fixed Effect Estimate = .05, 95% CI[.03, .07]). This indicates that negative emotion dysregulation was a significant predictor of PTSD symptoms, though there was significant variation in this cross-lagged effect, indicating that there was variability in this cross-lagged effect across participants. There was no significant fixed cross-lagged effect for PTSD symptoms and next-interval negative emotion dysregulation. However, there was a random effect for this cross-lagged effect, indicating that there was variability in this cross-lagged effect across participants. Additionally, there were significant fixed and random autoregressive effects for both negative emotion dysregulation and PTSD symptoms, suggesting that both processes had a spill-over effect from the previous assessment that varied across individuals. Furthermore, random means and random innovations (i.e., variances) were significant and these parameters also randomly varied across individuals, suggesting variability in the mean, inertia, and variability of negative emotion dysregulation and in the mean, inertia, and variability of PTSD symptoms across individuals.

The correlations among random effects are presented in Table 3. Individuals with higher negative emotion dysregulation also had more PTSD symptoms, spill-over effects for negative emotion dysregulation, and a higher residual variance for negative emotion dysregulation. Individuals with PTSD symptoms associated with next-interval negative emotion dysregulation also reported more negative emotion dysregulation and PTSD symptoms. Spill-over effects for negative emotion dysregulation was associated with spill-over effects for PTSD and the residual variance for PTSD. Spill-over effects for PTSD was associated with PTSD symptoms associated with next-interval negative emotion dysregulation. The residual variance for PTSD was associated with the residual variance for negative emotion dysregulation.

### Positive Emotion Dysregulation

After model testing, a model in which random effects were estimated for the means, autoregressions, cross-lags, and residual variances (but no random effect for the covariance) converged with PSR values of 1.002 after 5,000 iterations. The fixed and random effects are presented in Table 2. No significant fixed cross-lagged effects emerged. However, there were significant random effects for both cross-lagged effects. This indicates that there was significant variability in these cross-lagged effects across participants. A significant residual covariance suggested that positive emotion dysregulation and PTSD tended to co-occur and that the magnitude of the effect varied across people. Furthermore, there were significant fixed and random autoregressive effects for both positive emotion dysregulation and PTSD symptoms, suggesting that both processes had a spill-over effect from the previous assessment that varied across individuals. Moreover, the random means and random innovations (i.e., variances) were significant and these parameters also randomly varied across individuals, suggesting variability in the mean, inertia, and variability of positive emotion dysregulation and in the mean, inertia, and variability of PTSD symptoms across individuals.

The correlations among random effects are presented in Table 3. Individuals with higher positive emotion dysregulation had more PTSD symptoms and a higher residual variance for positive emotion dysregulation. Spill-over effects for positive emotion dysregulation was associated with spill-over effects for PTSD. Positive emotion dysregulation associated with next-interval PTSD symptoms was associated with PTSD symptoms associated with next-interval positive emotion dysregulation. The residual variance for PTSD was associated with the residual variance for positive emotion dysregulation.

## Discussion

The current study is the first to examine the reciprocal daily associations between both negative and positive emotion dysregulation and PTSD symptoms. Results at the between-person level indicated that women who had higher negative and positive emotion dysregulation also had more PTSD symptoms. At the within-person level, findings supported a significant contemporaneous effect between positive emotion dysregulation and PTSD symptoms, indicating that positive emotion dysregulation co-occurred with PTSD symptoms. Moreover, the cross-lagged effect from negative emotion dysregulation to next-interval PTSD symptoms was significant, suggesting that earlier negative emotion dysregulation predicted later PTSD symptoms. However, inconsistent with our study hypotheses, a contemporaneous effect between negative emotion dysregulation and PTSD symptoms was not detected. Further, cross-lagged effects were not found from positive emotion dysregulation to next-interval PTSD symptoms or from PTSD symptoms to next-interval negative and positive emotion dysregulation. Collectively, these findings underscore important next steps for research and clinical practice focused on emotion dysregulation and PTSD among women experiencing IPV.

Evidence for the predictive role of negative emotion dysregulation on PTSD symptoms aligns with the literature to suggest that emotion dysregulation contributes to the maintenance and exacerbation of PTSD symptoms. Specifically, individuals who experience elevated emotion dysregulation may have limited access to effective strategies for managing trauma-related symptoms and distress (Tull et al., 2007; Weiss et al., 2013). Consequently, they may utilize avoidance in this context, which despite temporarily abating trauma-related symptoms and distress, has negative long-term consequences (Hayes et al., 1996), such as maintaining and/or exacerbating PTSD (Krause et al., 2008). Indeed, avoidance interferes with the processing of trauma memories, habituation to distressing emotions associated with trauma memories, and extinction of trauma-related fear responses, all of which underlie the development and subsequent continuation of PTSD symptoms following trauma exposure (Foa & Kozak, 1986). Therefore, our findings provide additional support for negative emotion dysregulation as a clinical target among women experiencing IPV. For instance, results here underscore the potential utility of measuring negative emotion dysregulation among women identified by experiences of IPV as a means of detecting those at greatest risk for developing and subsequently maintaining PTSD. Further, our findings provide support for addressing negative emotion dysregulation in the prevention and treatment of PTSD in this population. Indeed, there is growing evidence for the utility of targeting negative emotion dysregulation in psychological treatments (Gratz et al., 2015), including for the treatment of PTSD (Cloitre et al., 2002; Harned et al., 2014). Future research is necessary to evaluate the effectiveness of these treatments among women experiencing IPV. More broadly, this knowledge may be utilized to inform ecological momentary interventions to be delivered in real-time to prevent PTSD symptom exacerbation.

Inconsistent with study hypotheses, significant cross-lagged effects were not found from positive emotion dysregulation to next-interval PTSD symptoms or from PTSD symptoms to next-interval negative and positive emotion dysregulation. A few potential conclusions can be drawn from these findings. First, our results further specify the directionality of the association between negative emotion dysregulation and PTSD: earlier negative emotion dysregulation was related to later PTSD symptoms, but earlier PTSD symptoms were not related to later negative emotion dysregulation. These findings suggest that the relation between negative emotion dysregulation and PTSD may not be bidirectional, despite theoretical accounts that suggest otherwise, or at least not across this time period. Second, in terms of positive emotion dysregulation specifically, evidence for significant contemporaneous, but not cross-lagged effects, suggests that while positive emotion dysregulation and PTSD symptoms co-occur, they may not proximally predict one another within the window of time specified here. That is, when individuals are experiencing PTSD symptoms, they also are experiencing elevated levels of positive emotion dysregulation. However, our results did not indicate that these individuals’ levels of positive emotion dysregulation were directly related to their later PTSD symptoms or vice versa. Notably, methodological factors may explain the lack of significant cross-lagged effects. For instance, the amount of time between the surveys may have been too long to capture the causal processes between positive emotion dysregulation and PTSD symptoms as well as from PTSD symptoms to negative emotion dysregulation. As one example, it may be that increases in negative emotion dysregulation are associated with PTSD symptoms that occur more proximal to the negative emotion dysregulation, such as within minutes. Future investigations are needed to examine these associations using shorter lags. Another possibility is that only a subset of individuals may exhibit cross-lagged effects between positive emotion dysregulation and PTSD symptoms as well as from PTSD symptoms to negative emotion dysregulation. Future research using idiographic approaches may identify individuals for whom negative and positive emotion dysregulation drive PTSD symptoms and PTSD symptoms drive negative and positive emotion dysregulation.

It is imperative to consider study limitations when interpreting the findings. First, though acceptable, survey compliance was slightly lower than other studies using ESM (Collins et al., 2003; Searles et al., 2002; Searles et al., 1995). This is likely because we did not exclude women with current substance use disorders or who were unstably housed, as other studies have. Indeed, our ESM compliance rate is comparable to other studies that have used similar inclusion and exclusion criteria (Sullivan et al., 2020; Sullivan et al., 2016). Second, consistent with recommendations in the literature for collecting intensive longitudinal data from women in relationships characterized by IPV (Sullivan et al., 2011), non-random surveys were scheduled in the morning, afternoon, and evening to capture the entire day while simultaneously providing women with greater control over when they completed surveys (e.g., answered questions about IPV). One limitation of this approach is that women who initiated calls may have done so in systematic ways (e.g., based upon their schedule, emotions, or activities). Relatedly, we chose to use a potentially more discrete method of ESM data collection—IVR—to protect women from any risk associated with their partner discovering they were in a study about their relationship, such as noticing a study app on their phone. IVR is a reliable and valid method for collecting ESM data (Perrine et al., 1995; Schroder & Johnson, 2009). Third, our findings cannot be assumed to generalize to non-IPV populations, and thus require replication across more diverse samples of individuals who experience IPV (e.g., women recruited from shelters, men, people in same-sex relationships). Further, as was noted earlier, our models were somewhat constrained by the within-day reporting windows we chose. Effects might unfold across shorter intervals and/or might vary in terms of the lag duration. Future research using more fine-grained reporting strategies (such as ecological momentary assessment) could further investigate this possibility.

Despite these limitations, our results advance literature on the reciprocal daily relations between both negative and positive emotion dysregulation and PTSD symptoms. Specifically, findings provide support for the co-occurrence of positive emotion dysregulation and PTSD symptoms as well as a cross-lagged effect from negative emotion dysregulation to PTSD symptoms. Future research in this area (e.g., shorter lags, idiographic approaches) will inform efforts to detect and intervene on PTSD symptoms among women experiencing IPV.

## Figures and Tables

**Table 1.** Sample Demographics and Descriptive Statistics

	*M* (*SD*)	Range	*n* (%)
Age	40.66 (11.61)	19 – 65	
Racial/Ethnic Background			
Black or African American			45 (31.0%)
White			59 (40.7%)
American Indian/Alaska Native			12 (8.3%)
Hispanic or Latina			17 (11.7%)
Multiracial			8 (5.5%)
Not listed ^a^			3 (2.1%)
Prefer not to respond			1 (0.7%)
Years of Education Completed	12.45 (2.00)	8 – 18	
Employment			
Full time (35+ hours per week)			7 (4.8%)
Part time (<35 hours per week)			17 (11.7%)
Unemployed			92 (63.4%)
Not in labor force			20 (13.8%)
Prefer not to respond			9 (6.2%)
Monthly Household Income	$1,529.11 ($2,078.03)	$0 - $10,416.67	
Relationship Status			
Married			14 (9.7%)
Unmarried			108 (74.5%)
Separated or divorced			12 (8.3%)
Prefer not to respond			11 (7.6%)
Relationship Length (in years)	5.48 (5.30)	0.5 – 24	
Days with Partner Per Week	5.87 (1.82)	0 – 7	
Current PTSD Diagnosis			53 (36.6%)

*Note.* Sample size is 145 participants. Percentages presented are valid percentages; ^a^Of the participants who indicated that their racial background was not listed, one self-described themselves as Cape Verdean, one as Portuguese, and one reported being unsure of their racial background. PTSD = posttraumatic stress disorder.

**Table 2.** Unstandardized fixed and random effects from model examining PTSD and Emotion Dysregulation

Parameter	Fixed effects (means)			Random effects (variances)		
	Est	95% CI: Lower	95% CI: Upper	Est	95% CI: Lower	95% CI: Upper
*Negative Emotion Dysregulation*						
Neg intercept	5.80*	5.07	6.53	18.82*	14.68	24.68
PTSD intercept	3.00*	2.68	3.30	3.83*	3.00	5.00
Autoregressive effects						
Neg(t-1) --> Neg(t)	0.25*	0.21	0.30	0.06*	0.04	0.08
PTSD(t-1) --> PTSD(t)	0.21*	0.16	0.25	0.05*	0.04	0.07
Cross-lagged effects						
PTSD(t-1) --> Neg(t)	0.04	−0.01	0.10	.04*	0.02	0.07
Neg(t-1) --> PTSD(t)	0.04*	0.02	0.06	0.002*	0.001	0.01
Neg residual variance	1.73*	1.39	2.08	4.22*	3.32	5.50
PTSD residual variance	0.68*	0.51	0.86	1.05*	0.82	1.37
*Positive Emotion Dysregulation*						
Pos intercept	5.62*	4.89	6.33	17.74*	13.92	23.11
PTSD intercept	3.002*	2.67	3.33	3.79*	2.98	4.90
Autoregressive effects						
Pos(t-1) --> Pos(t)	0.25*	0.21	0.30	0.05*	0.04	0.07
PTSD(t-1) --> PTSD(t)	0.22*	0.17	0.27	0.05*	0.04	0.07
Cross-lagged effects						
PTSD(t-1) --> Pos(t)	0.05	−0.002	0.10	0.02*	0.01	0.04
Pos(t-1) → PTSD(t)	0.01	−0.01	0.03	0.002*	0.001	0.01
Pos residual variance	1.66*	1.36	1.95	3.10*	2.41	4.04
PTSD residual variance	0.68*	0.50	0.85	1.06*	0.83	1.39

*Note.* Sample size is 145 participants. PTSD = posttraumatic stress disorder. Neg = negative. Pos = positive.

* indicates that the 95% credibility interval does not contain 0.

**Table 3.** Standardized between-person correlations among random effects of VAR DSEMs for model examining PTSD

Parameter	1	2	3	4	5	6	7	8
1. Emotion Dysregulation (ED) intercept	--	.34*	.08	.002	−.21	−.33	.53*	.09
2. PTSD intercept	.60*	--	.05	.05	−.04	−.20	.33*	.03
3. Autoregressive effect ED(t-1) --> ED(t)	.25*	.17	--	.38*	.05	−.24	.11	−.10
4. Autoregressive effect PTSD(t-1) --> PTSD(t)	.03	.05	.37*	--	.08	−.03	−.04	−.02
5. Cross-lagged effect PTSD(t-1) --> ED(t)	.22	.36*	.16	.56*	--	.46*	−.01	.30
6. Cross-lagged effect ED(t-1) --> PTSD(t)	−.50*	−.23	−.03	.01	.19	--	−.56*	−.16
7. ED residual variance	.43*	.34*	.15	.11	.07	−.61	--	.35*
8. PTSD residual variance	−.02	.04	−.17	−.04	−.13	.05	.41*	--

*Note.* Sample size is 145 participants. PTSD = posttraumatic stress disorder. Results from model assessing positive emotion dysregulation (ED) depicted on upper triangle. Results from model assessing negative emotion dysregulation (ED) depicted on lower triangle.

## Data Availability

Data available on request.
